# Nationwide Data on the Characteristics of Linked-to-Care Chronic Hepatitis B in Korea

**DOI:** 10.3390/jcm10204633

**Published:** 2021-10-09

**Authors:** Young Cheol Ju, Dae Won Jun, Eileen L. Yoon, Sang Bong Ahn, Yun Jin Kim, Mindie H. Nguyen

**Affiliations:** 1 Department of Translational Medicine, Graduate School of Biomedical Science and Engineering, Hanyang University, Seoul 04763, Korea; feenswimmer@gmail.com; 2 Department of Internal Medicine, Hanyang University Hospital, Hanyang University College of Medicine, Seoul 04763, Korea; 3 Nowon Eulji Medical Center, Department of Internal Medicine, Eulji University College of Medicine, Seoul 01830, Korea; dr486@eulji.ac.kr; 4 Biostatistical Consulting and Research Laboratory, Medical Research Collaborating Center, Hanyang University, Seoul 04763, Korea; yeun0148@hanyang.ac.kr; 5 Division of Gastroenterology and Hepatology, Stanford University Medical Center, Palo Alto, CA 94305, USA; mindiehn@stanford.edu

**Keywords:** chronic hepatitis B, public health, delivery of health care, diagnosis rate, hepatocellular carcinoma

## Abstract

Linkage-to-care rate of chronic hepatitis B (CHB) is less well characterized. We aimed to evaluate the proportion, characteristics of CHB patients who are linked to care. We retrospectively analyzed insurance reimbursement claims data provided by the Korean National Health Insurance Service. CHB patients who had at least two clinic or hospital visits that were associated with a CHB diagnostic code during 2002–2006 were included. Those without a history of malignancy at baseline were followed up until 2018. Mean follow-up period was 14.5 ± 2.9 years. Among the participants, 553,085 patients (35.8%) were found to be linked to care. The rates were lower in men than women (35.7% vs. 36.0%, *p* = 0.006). By age, it was highest for the 40′s age group at 44.8% and lowest at 29.4% for the 20′s age group (All *p* < 0.0001). The linkage-to-care rate was higher in rural area than metropolitan area (*p* < 0.0001). The 15-year cumulative incidence of hepatocellular carcinoma and overall survival rates among linked-to-care CHB patients were 18.2% and 93.8%, respectively. Two thirds of CHB patients were not linked to care. Those who are male, dwelling in metropolitan areas, and not in life transition periods need to be targeted to improve the linkage-to-care rate in Korea.

## 1. Introduction 

Chronic hepatitis B (CHB) is a major global public health problem affecting approximately 257 million people worldwide. Therefore, the World Health Organization has set a goal to eliminate viral hepatitis by 2030. However, this ambitious goal would require a diagnosis rate of 90% or more and a treatment rate of 80% or more [1]. Meanwhile, only approximately 10.5% (27 million) of the global CHB population are estimated to be aware of their infection; and among the suitable candidates for treatment, only 16.7% (4.5 million) may have received antiviral agents [2].

CHB prevalence, diagnosis rate, and ‘linkage-to-care (LTC)’ rate vary according to regions and nations [3]. According to the Korean National Health and Nutrition Examination Survey (KNHANES), the prevalence of CHB in Korea as of 2008 was 3.5% [4,5]. The diagnosis rate for CHB is relatively high, at about 85% as estimated by a recent modeling study [3]. However, as in many other countries, the LTC rate of CHB in Korea has not been well characterized and may vary according to sex, income level, and region [2].

As social awareness and maintenance of a reasonable LTC rate are important steps towards effective control of viral hepatitis, it is important to determine the overall proportion of CHB patients who are linked to care. Additionally, LTC rates in relevant subgroups may provide information about target population that public health efforts should be sought. Therefore, we aimed to estimate the proportion of total CHB population who were linked to care in Korea using a large nationwide database.

## 2. Materials and Methods

### 2.1. Data Source

This study used insurance reimbursement claims data provided by the Korean National Health Insurance Service (NHIS). The health insurance claims data are generated when a health care provider submits a reimbursement claim to the NHIS for payment of the proportion of medical services provided to the patient that is covered by the NHIS. Korea achieved universal health coverage of its population in 1989; and all insurance plans were merged into a single payer system in 2000 [6]. Health care providers in Korea are all automatically eligible, and are obliged to treat patients for services covered under the system. The system is administered by the Ministry of Health and Welfare. In 2018, 97.2% of the Korean population was covered under the NHIS system [7].

### 2.2. Study Design

This was a retrospective longitudinal cohort study using the NHIS database data from 2002 to 2018. The initial 5-year period (baseline period), from 2002 to 2006, was used to identify the study population, and the period from 2007 to 2018 was used for long-term follow-up of this selected population. The provided data were approved by the NHIS following a review process, and the data were released with an encrypted number according to the disclosure principle. The study was conducted following approval from the institutional review board (IRB approval: HYUH 2018-08-003). Informed consent from study participants were waived owing to its historical cohort nature of the study using National Health Insurance data.

### 2.3. Definition of ‘Linked-to-Care’ CHB 

The inclusion criteria of this study were having a CHB diagnosis. The operational definition of LTC in CHB requires patients to have had at least two clinic or hospital visits associated with the diagnosis code B18.0 (chronic viral hepatitis B with delta-agent) or B18.1 (chronic viral hepatitis B without delta-agent) regardless of the major- or sub-diagnostic categories during the initial 5-year study period (from 2002 to 2006). Patients with missing region, income level, and follow-up information were excluded. 

### 2.4. Categorization of Variables 

The patient characteristics according to sex, age, region of treatment, income level, and major comorbidities at the inclusion time of the study population were investigated.

1. Age was categorized into seven categories: 0–19, 20–29, 30–39, 40–49, 50–59, 60–69, and ≥70 years.
2. Sex was categorized into male and female.
3. The region of the medical institution visited by patients was categorized into 16 categories: Seoul, Busan, Daegu, Incheon, Gwangju, Daejeon, Ulsan, Gyeonggi, Gangwon, Chungbuk, Chungnam, Jeonbuk, Jeonnam, Gyeongbuk, Gyeongnam, and Jeju. The region was also categorized into two groups: Metropolitan (cities with over one million residents) and other (cities/towns with less than one million residents and rural areas).
4. Level of income was categorized according to the 20-tier income levels used by the NHIS to determine the amount of medical insurance fee patients paid for medical services and goods. As the income level increases, the insurance fee payment increases. The poor are exempted from cost-sharing at the point of service, and the vulnerable patient groups have access to discounted copayment rates. Both of these groups are classified as the ‘0′ group. For the analysis, we classified patients into three groups: Lower (0–10 tier group), middle (11–16 tier group), and the upper one-third (17–20 tier group).

### 2.5. Other Operational Definitions

1. The comorbidities of special interest for the current study were diabetes, hypertension, dyslipidemia, coronary heart disease, chronic kidney disease, and cancer. The cancer comorbidities included in this study were thyroid, gastric, colorectal, lung, breast, prostate, and liver cancer.
2. Hepatocellular carcinoma (HCC) was defined by KCD-7 code “C22.0 (malignant neoplasm of liver cell carcinoma)” among the KCD code “C22 (malignant neoplasm of the liver and intrahepatic bile ducts)” or any of its sub-categories: C22.0, C22.1 (malignant neoplasm of intrahepatic bile duct carcinoma), C22.2 (malignant neoplasm of hepatoblastoma), C22.3 (malignant neoplasm of angiosarcoma of the liver), C22.4 (malignant neoplasm of other sarcomas of the liver), C22.7 (malignant neoplasm of other specified carcinomas of the liver), C22.9 (malignant neoplasm of the liver, unspecified). The current study used “C22.0” to investigate the incidence of HCC.
3. Survival was defined as transplant-free survival and was based on the NHIS code for death (clinical outcome code DTH_ASSMD_DT “4: Death”) and on codes of post-transplant medications and treatment code of the medical cost and treatment since there was no specific KCD 7 code for liver transplant.

### 2.6. Follow-Up

The mortality and development of HCC was investigated through follow-up observation until December 2018. Patients with a history of malignancy at baseline (2002–2006) were excluded from the longitudinal analysis of this study. 

### 2.7. Estimates of the “Linked-to-Care” CHB Proportion

**Figure 1:**
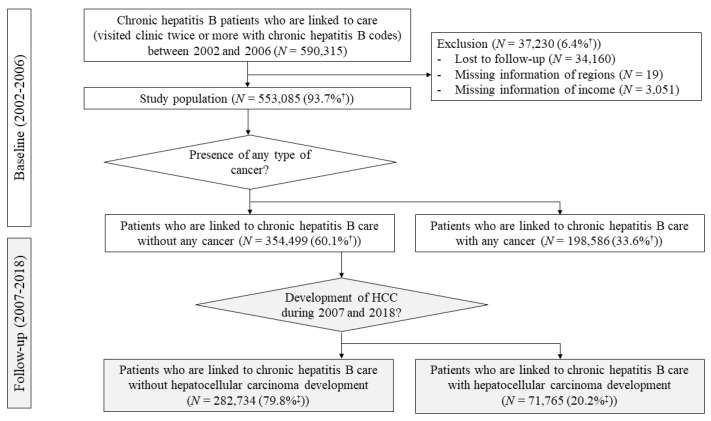
Flow diagram of the study population. CHB patients who were linked to care between 2002 and 2006 were identified at baseline. Those without any type of cancer were followed-up during 2007 and 2018. Twenty percent of linked-to-care CHB patients without any type of cancer showed hepatocellular carcinoma development during the follow-up period. The estimation of the linked-to-care population between 2002 and 2006 was based on the estimated reference chronic hepatitis B (CHB) data in 2005 in Korea. ^†^ Percentage was calculated as number of corresponding patients out of total linked-to-care CHB patients (*N* = 590,315). ^‡^ Percentage was calculated as number of corresponding patients out of linked-to-care CHB patients without any type of cancer at baseline (*N* = 354,499).

The total number of CHB patients in Korea (denominator, Figure 1) was obtained by multiplying the HBsAg seropositive prevalence data from The KNHANES for 2005 by the total 2005 population of Korea derived from the Korean Statistical Information Service (KOSIS) database and the Statistics Korea (KOSTAT) [8,9]. To obtain the proportion of LTC rate in CHB (numerator, Figure 1), we divided our total CHB population who are linked to care identified by the NHIS database during 2002–2006 by the total number of CHB patients in Korea derived from the KNHANES and KOSTAT for the year 2005. KNHANES and KOSIS data were also used to calculation of subgroup data. 

### 2.8. Statistical Analysis

Descriptive statistics were applied to characterize linked-to-care CHB patients using NHIS population parameters. Probability statistical analyses were applied for review of the difference between groups by age, sex, region, and income level. Kaplan-Meier analysis was used for survival analysis, and log-rank test was used for the analysis of differences between the groups. Statistical significance was defined with two-sided *p* value of <0.05. Statistical analysis was performed using SAS software (version 9.4; SAS Institute, Inc., Cary, NC, USA).

## 3. Results

### 3.1. Selection and Characteristics of CHB Patients Who Are Linked to Care

Of the CHB patients registered in NHIS between 2002 and 2006, a total of 590,315 patients were identified as linked-to-care CHB patients who visited clinic or hospital twice or more associated with chronic hepatitis B diagnosis codes (Figure 1). Of these, 34,160 patients without any reimbursement claim data during the follow-up periods (2007–2018) and 3070 patients without information about region (*N* = 19) or social economic (insurance fee) information (*N* = 3051) were excluded. Finally, 553,085 linked-to-care CHB patients with appropriate information were enrolled as the study population. 

The mean age of the cohort was 41 years old and about half resided in metropolitan area (Table 1). The male-to-female ratio was 1.36 (male, 57.6%), and male made up the majority in age groups younger than 59 years. The largest groups of linked-to-care CHB patients were those in their forties (27.5%), followed by those in their thirties (22.3%) then fifties (16.9The most common cancer was liver cancer (88,173 patients; 15.9% of the entire study population and 44.4% of all cancers presented at baseline). The second and third most common cancers were colorectal cancer and gastric cancer at 1.5% and 1.4% of the entire study population, respectively. 

### 3.2. Proportion of Linked-to-Care CHB among the Total CHB Population 

**Figure 2:**
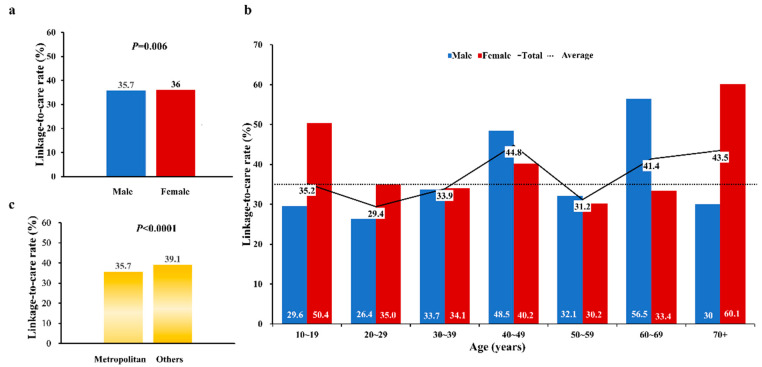
Estimated LTC rates of CHB patients between 2002 and 2006 according to (**a**) sex, (**b**) age groups, and (**c**) regions.

The estimated linked-to-care CHB rate was 35.8% (553,085 × 100(%)/1,545,377) based on the reported HBsAg seroprevalence rate and the total population estimates for the year of 2005 in Korea (Figure 1). The LTC rate was significantly lower in men (35.7%) than in women (36.0%) (*p* = 0.006) (Figure 2). By age, the LTC rate was highest for the 40s age group at 44.8% and lowest at 29.4% for the 20′s age group (Figure 2). Among males, the LTC rate of the 60s age group was higher than that of the 40s age group (*p* < 0.00001). For females, the LTC rate of the above 70s age group was the highest, at 60.1%, followed by the 10s (50.4%), and 40s (40.2%) age groups. Those rates were significantly lower in the metropolitan areas than the other areas (35.7% vs. 39.1%, *p* < 0.0001). Notably, on average, each subscriber visited a clinic or hospital 17 times per year and 85 times during the 5-year study observation period to assess CHB LTC rates (data not shown). Only 6.5% did not visit a clinic or hospital in a 1-year period. 

### 3.3. Long-Term Outcomes of Linked-to-Care CHB Patients in Korea

**Figure 3:**
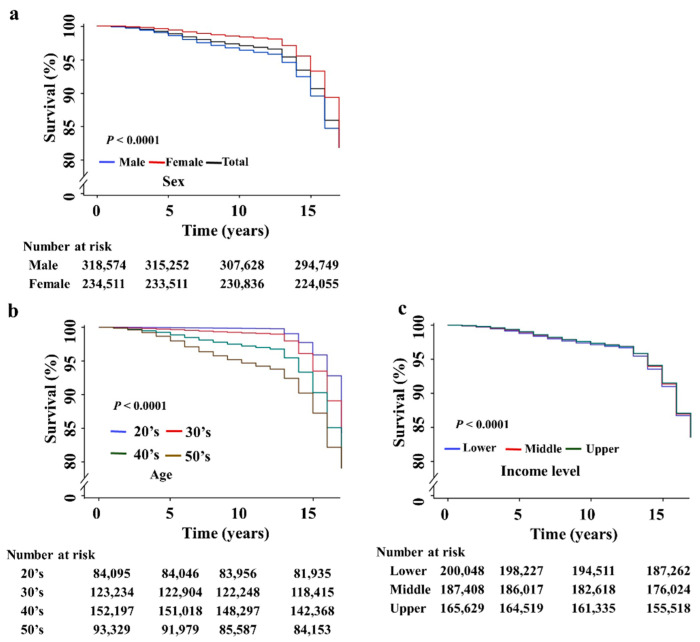
Survival rates of linked-to-care CHB patients according to (**a**) sex, (**b**) age, and (**c**) income level. All the *p*-values were less than 0.0001 in the comparison between 20’s vs. 30’s, 20’s vs. 40’s, 20’s vs. 50s, 30’s vs. 40’s, 30’s vs. 50’s, and 40’s vs. 50’s.

The mean observation period was 14.5 ± 2.9 years. Overall, cumulative 5, 10-, and 15-year survival rates were 99.2%, 97.4%, and 93.8%, respectively. The 5-, 10-, and 15-year survival rates for males were 99.0%, 96.6%, and 92.5% as compared to 99.2%, 97.4%, and 93.8% for females, respectively (*p* < 0.0001) (Figure 3a). Survival rates decreased significantly by age (Figure 3b). Survival rate in their 50′s of CHB was the lowest compared to those in their 20′s, 30′s, and 40′s (all *p* < 0.0001) The survival rates appeared similar for the three income groups, with the 15-year rates of 93.9%, 93.9, 93.9% for the upper, middle, and lower tertile groups, respectively although the *p*- value was lower than 0.0001 (Figure 3c).

### 3.4. HCC Incidence among Linked-to-Care CHB Patients and Their Survival 

**Figure 4:**
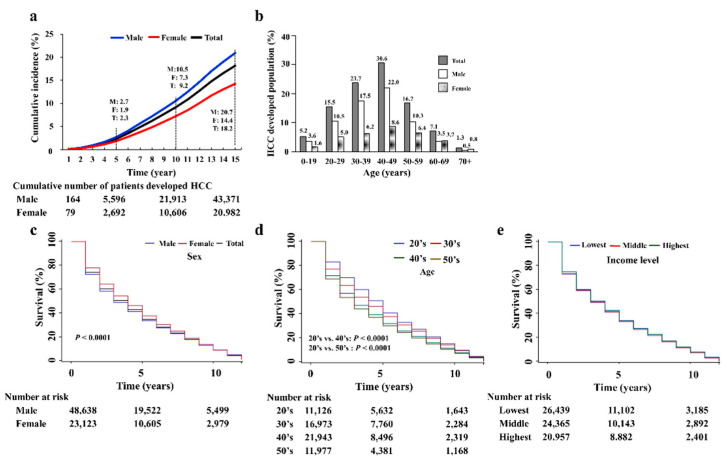
Cumulative incidence rate and survival rates of patients who newly developed HCC during LTC. (**a**) Cumulative incidence of HCC development according to sex. (**b**) The distribution of age group who developed HCC during LTC. (**c**) Kaplan-Meier plots for survival estimates after HCC development by sex. (**d**) Kaplan-Meier plots for survival estimates after HCC development by age. All the *p*-values were less than 0.0001 in the comparison between 20’s vs. 30’s, 20’s vs. 40’s, 20’s vs. 50s, 30’s vs. 40’s, 30’s vs. 50’s, and 40’s vs. 50’s. (**e**) Kaplan-Meier plots for survival estimates after HCC development by income levels.

Among the 553,085 study population, 354,499 (63.5%) subjects did not have any history of malignancy at baseline (2002–2006) and they were included in the incident HCC analysis. During the follow-up period (2007–2018), 71,765 patients developed new HCC over a mean follow-up of 14.5 years (Figure 1). Cumulative incidence for HCC development at 15 years was significantly higher in male (male vs. female, 20.7 vs. 14.4, *p* < 0.0001). (Figure 4a). By age, the 40s age group had the highest proportion of patients who developed HCC while linked-to-care (30.6%), followed by the 30s group (23.7%), with also higher rates among males (Figure 4b).

Survival among patients who developed HCC was higher in women as compared to men (*p* < 0.001) (Figure 4c). The assessment of survival rates of the newly HCC developed linked-to-care CHB patients in their 20s, 30s, 40s, and 50s showed decreasing rates with age, lowest in their 20′s and the lowest in their 50′s (*p* < 0.0001) (Figure 4d). The overall differences in survival rate among the age groups decreased as time passed. There was no significant difference in the survival rates among the groups according to income level (lower vs. middle, *p* = 0.4524; lower vs. upper, *p* = 0.5220; middle vs. upper, *p* = 0.922) (Figure 4e).

## 4. Discussion

Our study found only 35.8% of the CHB population of South Korea are linked to care, despite using a rather “liberal” definition of LTC, which only requires two clinic or hospital visits with a CHB diagnosis code in any position during a 5-year study period. Thus, in South Korea where universal healthcare coverage is well established, the majority (64.2%) of CHB patients are not linked to care, i.e., not getting any care at all or in very suboptimal. In addition, this is most likely a gross underestimation of the rate of optimal LTC rate because most professional society guidelines recommend monitoring of liver function and HBV DNA every 3–6 months and HCC surveillance with imaging ± serum biomarker test every 6 months [10,11]. We believe that all the CHB care during the 5-year observation period would have been captured because any CHB-related tests such as HBeAg, HBV DNA RT-PCR, and AFP, would have to be ordered with CHB diagnostic codes to be reimbursed, during our review of all the major and minor diagnostic codes. It is also important to note that our study cohort visited clinics/hospitals very often (only 6.5% did not go to clinics/hospital in one year, data not shown), but they just did not receive care regarding CHB. Taken together, we could assume that healthcare coverage and access to medical service system may not be the major causes for lack of CHB care in Korea, which is also supported by the lack of survival difference observed in our study among CHB patients who developed HCC by income levels and by geographic regions (metropolitan vs. smaller cities/towns/rural areas). 

Though prior studies from the U.S. have reported that male sex and higher income were independently associated with higher compliance with health care utilization among subscribers of private medical insurance plans [12], we found that CHB showed higher rate of LTC in female than male in Korea. Nevertheless, males had higher incidence of HCC and poor survival rate after HCC development compared to females over 15 years in our study. Similarly, the lower LTC rates among the 20s and 30s age groups (29.4% and 33.7%) deserve further discussion because CHB care in these younger age groups would provide opportunities for preventive and surveillance care to lower the HCC incidence, which affected the 40s age group the most in our study. The lower survival in CHB patients who developed HCC in males and in the older groups also advocate for more targeted CHB intervention for these groups, so disease complications can be prevented before advancing age and comorbidity set in [13].

Our study has the following limitations. First, the operational definition of LTC rate was arbitrary. Although testing with HBV-DNA or antiviral treatment for CHB is not permitted without B18.1 or B18.0 diagnosis codes in reimbursement system, sometimes physicians omit diagnostic codes in real life practice, thus underestimating the LTC rates. However, since our LTC rate definition is quite liberal, this is unlikely to change the direction of our study results and conclusions. Second, the operational definition of CHB was based on B18.1 or B18.0 diagnostic codes, and there may be differences in the actual diagnosis and the claim code input for re-imbursement claims. However, abdominal ultrasound and AFP blood tests are served free of charge every 6 months for CHB patients in Korea; therefore, B18.1 or B18.0 diagnostic codes are strictly regulated by the Korean government. Third, this study is a 12-year follow-up study of a cohort from 2002 to 2006, so this may not reflect the LTC rate CHB patients in the more recent time. Regardless, since our LTC rate is very liberal, temporal improvement would have to be quite drastic to change the direction of our study conclusion. Fourth, data on liver transplant-free HCC development could have captured the CHB-related effect on the HCC development, but we could not further analyze the data. Further studies would be needed on the issue. Fifth, the most interesting issue would be the comparison of characteristics and outcomes of those who were linked to care and who were not. However, we could not capture the records of those who were not linked to care, due to the nature of National Health Insurance claims data. Sixth, we have not considered the effect of provider’s knowledge. In addition to sex and age, a number of other factors such as lack of patient education as well as lack of provider knowledge have been reported as potential barriers to CHB care [14]. A recent population-based study from the U.S. reported higher rates of antiviral treatment in CHB patients with specialist care as compared to those monitored in general medical clinics [15]. Optimal evaluation of CHB patients to include HBV DNA, ALT, and HBeAg serology has also been reported to be lower among general medical clinic patients as compared to patients under specialist care [16]. Thus, it would be important for additional patient outreach and provider education. In addition, there should be system-based quality measures and reminder system to alert patient and care providers when CHB-related tests are due at intervals recommended by practice guidelines. These factors should be considered in the future study.

In summary, 64.2% of CHB Koreans are under inadequate care. Those who are male, dwelling in metropolitan, not in life-transitioning period need to be focused for improvement of LTC rate in Korean CHB population. Therefore, barriers to care other than financial coverage and access to care should be sought and addressed.

## Figures and Tables

**Table 1:** Baseline characteristics of linked-to-care chronic hepatitis B patients.

Characteristics		Total	Age (Years)						
			0–19	20–29	30–39	40–49	50–59	60–69	≥70
Proportion of the group among the total patients		100%	6.6%	15.2%	22.3%	27.5%	16.9%	9.2%	2.4%
Age (years)		41.3 ± 14.5	14.0 ± 5.3	24.7 ± 2.9	34.7 ± 2.9	44.3 ± 2.8	54.0 ± 2.8	63.6 ± 2.8	73.9 ± 3.7
Male (%)		57.6	61.2	58.8	62.1	59.7	54.1	47.2	37.8
Follow-up periods (years)		14.5 ± 2.9	15.0 ± 1.6	15.0 ± 1.7	15.0 ± 2.2	14.7 ± 2.8	14.1 ± 3.4	13.2 ± 3.3	10.9 ± 4.5
Residing in metropolitan area (%)		49.9	49.6	52.5	50.1	50.4	50.7	44.1	41.3
Cancer (%)	All types of cancer	35.9	0.8	3.2	7.1	11.7	7.8	4.3	1.0
	- Liver	15.9	0.4	1.5	3.2	5.1	3.6	1.8	0.4
	- Colorectal	1.5	0.0	0.0	0.2	0.4	0.4	0.3	0.1
	- Gastric	1.4	0.0	0.0	0.1	0.4	0.4	0.3	0.1
	- Prostate	1.1	0.0	0.0	0.1	0.2	0.4	0.3	0.1
	- Lung	0.8	0.0	0.0	0.1	0.2	0.2	0.2	0.1
	- Thyroid	0.5	0.0	0.0	0.1	0.2	0.1	0.1	0.0
	- Breast	0.4	0.0	0.2	1.3	3.4	1.9	0.9	0.1
Income level (%)	Lower third	36.2	31.2	49.5	37.1	30.0	35.4	36.2	31.7
	Middle third	33.9	35.6	27.0	42.1	33.8	29.6	33.6	28.1
	Upper third	29.9	33.2	23.5	20.7	36.2	35.0	30.2	40.2

Numbers were presented as either mean ± standard deviation or percentage of patients out of the total study population.

## Data Availability

The data presented in this study are available on request from the corresponding author.
